# Mutations altering the DNA binding domains of the human RAD52 protein exert distinct effects on homologous recombination repair in *Saccharomyces cerevisiae*

**DOI:** 10.1093/g3journal/jkaf282

**Published:** 2025-11-23

**Authors:** Glenn M Manthey, Elise W Wolf, Jason Xu, M Cristina Negritto, Renee A Bouley, Ruben C Petreaca, Adam M Bailis

**Affiliations:** Irell & Manella Graduate School of Biological Sciences, Beckman Research Institute of the City of Hope, Duarte, CA 91010, United States; Yale School of Medicine, Yale University, New Haven, CT 06510, United States; Department of Internal Medicine, Stanford University, Palo Alto, CA 94305, United States; Molecular Biology Program, Pomona College, Claremont, CA 91711, United States; Department of Chemistry and Biochemistry, The Ohio State University at Marion, Marion, OH 43302, United States; Department of Molecular Genetics, The Ohio State University at Marion, Marion, OH 43302, United States; Cancer Biology, The James Comprehensive Cancer Center, Columbus, OH 43210, United States; Department of Health Science & Clinical Practice, College of Health Professions, Thomas Jefferson University, 901 Walnut Street, Room 1100F, Philadelphia, PA 19107, United States

**Keywords:** HsRAD52, ssDNA binding, DNA double-strand breaks, homologous recombination repair, budding yeast

## Abstract

RAD52 is a conserved member of the homologous recombination repair (HRR) apparatus from yeast to humans. Mutating conserved amino acids in the internal and external DNA binding domains of the human RAD52 protein (HsRAD52) has discrete effects in vitro. Previous studies have shown that HsRAD52 supports multiple mechanisms of HRR in budding yeast, suggesting the utility of this model system for exploring the correspondence between losses of HsRAD52 function *in vitro* and their impact *in vivo*. We report that disrupting the internal and external DNA binding domains of HsRAD52 produced distinct effects on the repair of genomic DNA double-strand breaks (DSB) by conservative and non-conservative HRR in budding yeast, suggesting that these domains contribute to separate mechanisms *in vivo*. The further elucidation of the effects of perturbations in the structure and biochemical function of HsRAD52 in living systems will provide new insight into its ability to support DSB repair, cancer susceptibility as well as new avenues for targeting HRR-deficient cancers.

## Introduction

RAD52 is a structurally and functionally conserved component of the DNA repair apparatus from yeast to humans (Hanamshet et al. 2016). Originally identified as a key factor in the repair of ionizing radiation-induced DNA double-strand breaks (DSB) by a homologous recombination-dependent process in budding yeast (Resnick 1975; Resnick and Martin 1976), it was subsequently shown to be involved in a broad array of mitotic and meiotic mechanisms (Game et al. 1980; Malone and Esposito 1980; Prakash et al. 1980; Fishman-Lobell et al. 1992; Le et al. 1999; Pannunzio et al. 2008; Mazina et al. 2017). In contrast to its central role in homologous recombination repair (HRR) in budding yeast, RAD52 plays a non-essential role in mammalian cells (Rijkers et al. 1998; Stark et al. 2004; Kan et al. 2017). Importantly, loss of RAD52 in human cells with defects in the primary HRR machinery elicits synthetic lethality, indicating that RAD52 is part of a discrete, auxiliary apparatus (Feng et al. 2011).

The repair of chromosomal DSBs by HRR is complex as it can occur by multiple mechanisms that can be divided into those that primarily conserve and those that obligatorily rearrange genome structure (Agmon et al. 2009). RAD52 is a key factor in both conservative and non-conservative HRR in budding yeast cells (Rudin and Haber 1988; White and Haber 1990; Ozenberger and Roeder 1991; Liefshitz et al. 1995; Bosco and Haber 1998; Smith and Rothstein 1999) and while also critical for non-conservative repair in mammalian cells (Stark et al. 2004; Adamson et al. 2020) it is only required for conservative repair upon inactivation of the primary HRR apparatus (Feng et al. 2011; Lok et al. 2013).

The requirement for RAD52 in the repair of chromosomal DSBs by non-conservative HRR in both yeast and mammalian cells has been attributed to its capacity to propagate the annealing of homologous, single-stranded DNA (ssDNA) sequences (Mortensen et al. 1996), which appear flanking chromosomal DSBs following nucleolytic processing (Lin et al. 1984; White and Haber 1990). In vitro studies of the function of RAD52 in ssDNA annealing indicate that RAD52 monomers undergo self-association to form multimeric ring (Kagawa et al. 2002; Singleton et al. 2002; Saotome et al. 2018) or linear (Liang et al. 2024) structures that facilitate DNA binding and annealing (Kagawa et al. 2002, 2008; Saotome et al. 2018; Liang et al. 2024). Mutational analyses revealed discrete internal and external DNA binding domains on RAD52 multimers (Kagawa et al. 2002, 2008; Saotome et al. 2018; Liang et al. 2024) whose function is associated with ssDNA annealing (Kagawa et al. 2008; Saotome et al. 2018). A physical model for the function of RAD52 in ssDNA annealing in vitro postulates that annealing of complementary ssDNA molecules proceeds through the sequential binding and release from these DNA binding domains (Saotome et al. 2018).

Expression of the human *RAD52* gene in budding yeast has been used to study the function of wild-type and mutant human RAD52 (HsRAD52) proteins in HRR in an intact model organism (Manthey et al. 2017; Clear et al. 2020). HsRAD52 has been shown to accumulate progressively and stably at genomic sequences adjacent to DSBs, consistent with the requirement of this function for their repair by HRR (Manthey et al. 2017; Clear et al. 2020). This suggests that mutations that affect the capacity of HsRAD52 to bind to DNA in vitro might exert an effect on DSB repair by HRR in budding yeast. We report here that mutations disrupting the function of the internal and external DNA binding domains of HsRAD52 have distinct effects on DSB repair by conservative and non-conservative HRR, consistent with discrete roles for DNA binding by HsRAD52 in these repair mechanisms. Implications of these observations regarding the genetic control of DSB repair, cancer susceptibility, and the search for small molecule inhibitors of HsRAD52 in budding yeast are discussed.

## Materials and methods

### Yeast strains

All strains used in this study (Supplementary Table 4) were isogenic, and constructed, using established procedures (Sherman et al. 1986). Strains utilized to detect cellular proteins or determine frequencies of DSB-stimulated ectopic gene conversion (EGC) or recombination between non-tandem direct repeats (DRR) were haploid segregants of diploid strains that had been sporulated and dissected. Strains used in yeast two-hybrid analysis were constructed by transforming the appropriate plasmids into the yeast strain Y187 (Clontech, Mountain View, CA, USA).

### Plasmids

Plasmids used in this study were constructed (Supplementary Table 5) using established techniques (Sambrook 2001). Plasmids constructed for yeast two-hybrid analysis were derived from pGBT9, containing the *TRP1* selectable marker and sequences encoding the Gal4 DNA binding domain, and pGAD424, containing a *LEU2* selectable marker and sequences encoding the Gal4 transcription activation domain (Clontech, Mountain View, CA, USA). The plasmid pGBT9-HsRAD52, used to express a fusion of the Gal4 DNA binding domain to the N-terminus of HsRAD52, and pGAD424-HsRAD52, used to express a fusion of the Gal4 transcriptional activation domain to the N-terminus of HsRAD52 were described in a previous study (Manthey et al. 2017). Plasmids pGBT9-HsRAD52-R55A, pGBT9-HsRAD52-K152A, pGBT9-HsRAD52-K133A, and pGBT9-HsRAD52-K102A, K133A were constructed from pGBT9-HsRAD52 to express fusions of the mutant HsRAD52 proteins to the Gal4 DNA binding domain. Plasmids pGAD424-HsRAD52-R55A, pGAD424-HsRAD52-K152A, pGAD424-HsRAD52-K133A and pGAD424-HsRAD52-K102A, K133A were constructed from pGAD424-HsRAD52 to express fusions of the mutant HsRAD52 proteins to the Gal4 transcriptional activation domain.

### Cellular protein detection

FLAG epitope tagged wild-type and mutant HsRAD52 proteins were detected in yeast cells expressing the proteins using Western blot analysis as described previously (Manthey et al. 2017). Briefly, whole cell extracts were prepared from yeast cells disrupted with glass beads, the proteins separated on SDS-PAGE gels, and the proteins transferred to PVDF membranes. Signals corresponding to FLAG-tagged HsRAD52 and GAPDH (used for normalization) proteins were detected with anti-FLAG M2 (Sigma-Aldrich, St. Louis, MO, USA) and anti-GAPDH (Aviva Systems Biology, San Diego, CA, USA) primary antibodies, goat anti-mouse HRP-conjugated secondary antibody (Thermo Fisher Scientific, Waltham, MA, USA), chemiluminescent signal generation (Thermo Fisher Scientific), and visualization on X-ray film.

### DSB-stimulated EGC frequency determination

The frequency of repair by conservative homologous recombination in budding yeast of a HO endonuclease catalyzed DSB in the *his3-Δ3*′*-HOcs* allele at the *HIS3* locus on chromosome XV utilizing sequences from the *his3-ΔMscI* allele at the *LEU2* locus on chromosome III was determined as described previously (Manthey et al. 2017). One milliliter YPGL cultures were inoculated with single yeast colonies, incubated at 30 °C until reaching a density of 1–2 × 10^7^ cells/ml, 20% galactose added to a final concentration of 2%, and the cultures incubated for an additional four hours at 30 °C. Dilutions of the cultures were plated onto solid YPD medium to assess viability and synthetic complete medium lacking histidine to select for recombinants. Colonies were counted after incubating the plates at 30 °C for three days. Frequencies of EGC were calculated by dividing the number of His^+^ recombinants by the number of viable cells plated. Mean frequencies of EGC, 95% confidence intervals, and the t-statistics and *P* values for pairwise strain comparisons were calculated using Prism (GraphPad Software, Boston, MA, USA). Representative His^+^ recombinants were scored for gene conversion at the DNA level by Southern blot analysis.

### DSB-stimulated recombination between non-tandem direct repeats

The frequency of repair by non-conservative homologous recombination in budding yeast of a DSB between 3′- and 5′-truncated copies of the *HIS3* coding sequence flanking the *URA3*-marked plasmid YIp5 inserted into the *HIS3* locus on chromosome XV was determined as described previously (Maines et al. 1998; Clear et al. 2020). Single yeast colonies grown on solid complete synthetic medium lacking uracil were used to inoculate one milliliter YPGL cultures, incubated at 30 °C until reaching a density of 1–2 × 10^7^ cells/ml, 20% galactose added to a final concentration of 2% and cultures incubated for an additional four hours at 30 °C. Dilutions were plated onto solid YPD medium to determine viability and onto synthetic complete medium lacking histidine to select for recombinants. Colonies were counted after incubation at 30 °C for three days. Frequencies of DSB-stimulated direct repeat recombination (DSB-DRR) were determined by dividing the number of His^+^ recombinant colonies by the number of viable cells plated. Mean DRR frequencies, 95% confidence intervals and the t-statistics and *P* values for pairwise strain comparisons were calculated using Prism. Representative His^+^ recombinants were secondarily scored for deletion events by replica plating to synthetic complete medium lacking uracil and/or Southern blot analysis.

### Quantitating protein interaction by yeast two-hybrid analysis

Interaction between wild-type or mutant HsRAD52 proteins were assessed as previously described (Manthey et al. 2017). Plasmids pGBT9 and pGAD424 and their derivatives were transformed into the yeast strain Y187 and transformant colonies used to inoculate five milliliter cultures of synthetic complete medium lacking leucine and tryptophan and grown to saturation at 30 °C. Cell extracts were prepared and used to determine the specific activities of beta-galactosidase in Miller units. Mean specific activities, 95% confidence intervals and the t-statistics and *P* values for pairwise strain comparisons were calculated using Prism.

### Protein structure mutation analysis

Previously published X-ray structures (Saotome et al. 2018) were used as a template to model the impact of mutations on DNA binding to the internal and external binding sites (PDB 5XRZ and 5XS0, respectively). Mutated amino acids were introduced to each of the 11 monomers in the structure using the mutagenesis function in PyMOL (version 3.0.3). An electrostatic surface potential was generated for each protein without DNA using a PyMOL APBS Plug-In [PMID 11517324] to determine how the mutated amino acids affect the positive surface potential of the DNA binding sites.

### cBioPortal query

The curated set of non-redundant studies was used, which includes both PanCancer studies (including TCGA data) as well as other smaller studies. It excludes cell lines because the goal was to understand whether these mutations occur in primary cancers. An independent query with cell lines only did not reveal the presence of any of the mutations studied here. The cBioPortal mutual exclusivity calculator was used to determine the probability of co-occurrence in cancers of *HsRAD52* mutations with mutations in *BRCA1* or *BRCA2*. The calculator computes a probability value that is interpreted as a co-occurrence of mutations if *P* < 0.05.

## Results

### Expressing DNA binding mutant alleles of *HsRAD52* yields stable proteins in budding yeast

**Fig. 1.**
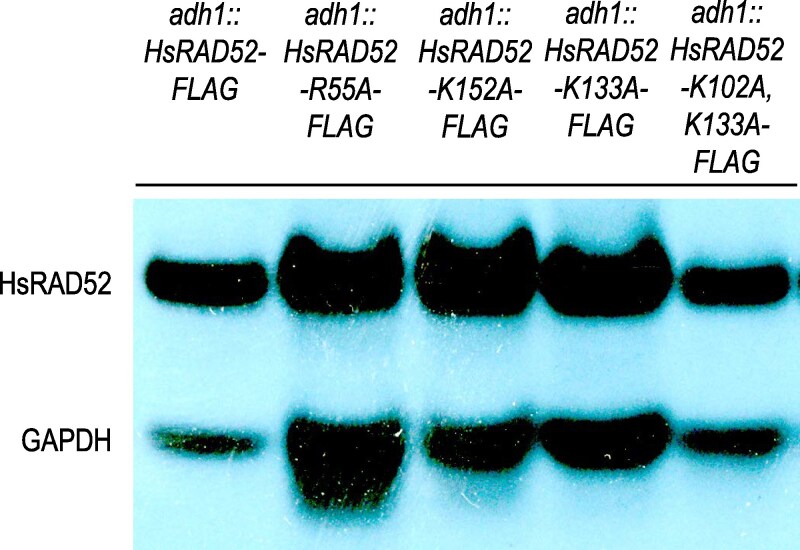
Expression of DNA binding mutant alleles of *adh1::HsRAD52-FLAG* yields stable proteins in budding yeast cells. Proteins from whole cell extracts from budding yeast strains expressing *adh1::HsRAD52-FLAG*, *adh1::HsRAD52-R55A-FLAG*, *adh1::HsRAD52-K152A-FLAG*, *adh1::HsRAD52-K133A-FLAG* and *adh1::HsRAD52-K102A, K133A-FLAG* were separated by SDS-PAGE, blotted to PVDF and probed with anti-FLAG and anti-GAPDH antibodies. Strain genotypes are listed across the top of the figure. Signals corresponding to wild-type and mutant HsRAD52, and GAPDH are labeled on the left side of the figure.

Current models for HsRAD52 function in DSB repair in vivo hypothesize a critical role for its DNA binding activity (Mortensen et al. 1996; Hanamshet et al. 2016; Manthey et al. 2017; Clear et al. 2020), suggesting that mutations in the *HsRAD52* gene that confer DNA binding defects *in vitro* might affect DSB repair *in vivo*. To investigate the potential role of DNA binding by HsRAD52 in DSB repair *in vivo* we used our established strategy for expressing *HsRAD52* in budding yeast (Manthey et al. 2017; Clear et al. 2020) to express four mutant alleles that affect DNA binding *in vitro* (Kagawa et al. 2002, 2008; Saotome et al. 2018), *HsRAD52-R55A, -K152A, -K133A,* and *-K102A, K133A*. As observed previously with strains expressing FLAG-tagged wild-type and mutant alleles of *HsRAD52* (Manthey et al. 2017; Clear et al. 2020), strains expressing the four DNA binding mutant alleles displayed a single, stable, 49-kDa species (Fig. 1).

### Mutations affecting DNA binding by HsRAD52 minimally affect self-association

**Fig. 2.**
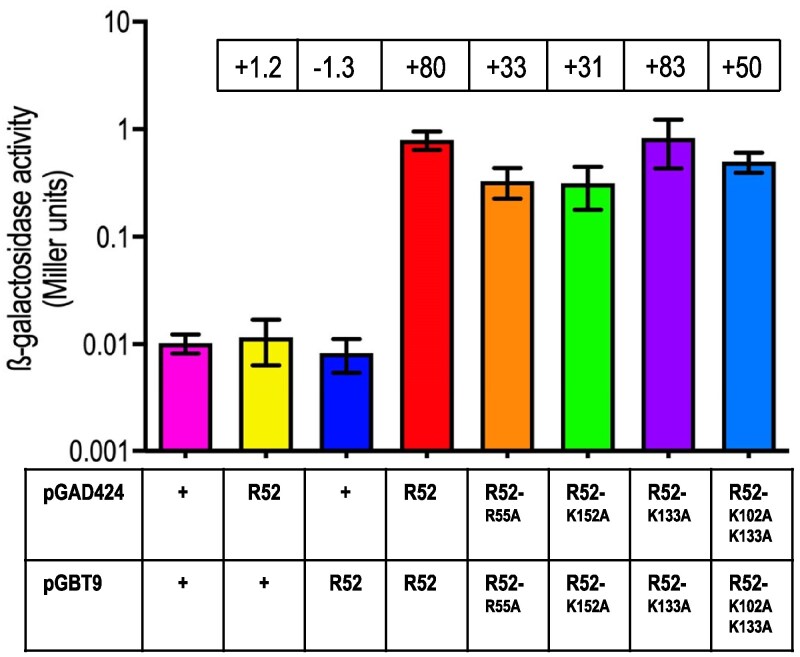
DNA binding mutant proteins display minimal loss of self-association in the yeast two-hybrid assay budding yeast strain Y187 was transformed with plasmids pGBT9 and pGAD424, or derivatives containing wild-type or mutant *HsRAD52* sequences. Transformants were grown and whole cell extracts prepared. β-galactosidase specific activities, in Miller units were determined with extracts from a minimum of 10 independent cultures of each transformant. Mean specific activities and 95% confidence intervals are plotted. Fold differences from the pGBT9/pGAD424 negative control appear in boxes above the plots of the mean specific activities.

HsRAD52 monomers self-associate to form higher order structures *in vitro* and *in vivo* that are thought to contribute to their role in DSB repair (Shen et al. 1996; Van Dyck et al. 1998, 1999; Stasiak et al. 2000; Kagawa et al. 2002; Saotome et al. 2018; Liang et al. 2024). Domain mapping of HsRAD52 has suggested that the area of the protein implicated in self-association (Shen et al. 1996) overlaps with regions attributed to DNA binding (Kagawa et al. 2001, 2002, 2008; Saotome et al. 2018). We investigated the potential effects of the *HsRAD52-R55A, -K152A, -K133A,* and *-K102A, K133A* DNA binding mutations on HsRAD52 self-association using yeast two hybrid analysis. Yeast cells transformed with plasmids expressing the Gal4-HsRAD52 DNA binding domain and Gal4-HsRAD52 transcription activation domain fusion proteins displayed levels of β-galactosidase activity that were substantially above background (80-fold; Fig. 2; Supplementary Table 1), consistent with a stable interaction between HsRAD52 monomers. Yeast cells transformed with the plasmids carrying the *HsRAD52-R55A, -K152A,* and *-K102A, K133A* mutant fusion plasmids displayed modestly but significantly reduced β-galactosidase activity levels (1.6- to 2.6-fold, *P* ≤ 0.0023), while yeast cells transformed with the *HsRAD52-K133A* mutant fusion plasmids displayed levels that were not significantly different from those transformed with the wild-type fusion plasmids (*P* = 0.87). These results indicate that these DNA binding mutations confer no change, or a modest loss of the capacity of HsRAD52 to self-associate in budding yeast.

### DNA binding mutations profoundly impair the capacity of HsRAD52 to repair a genomic DSB by conservative HRR in budding yeast cells

**Fig. 3.**
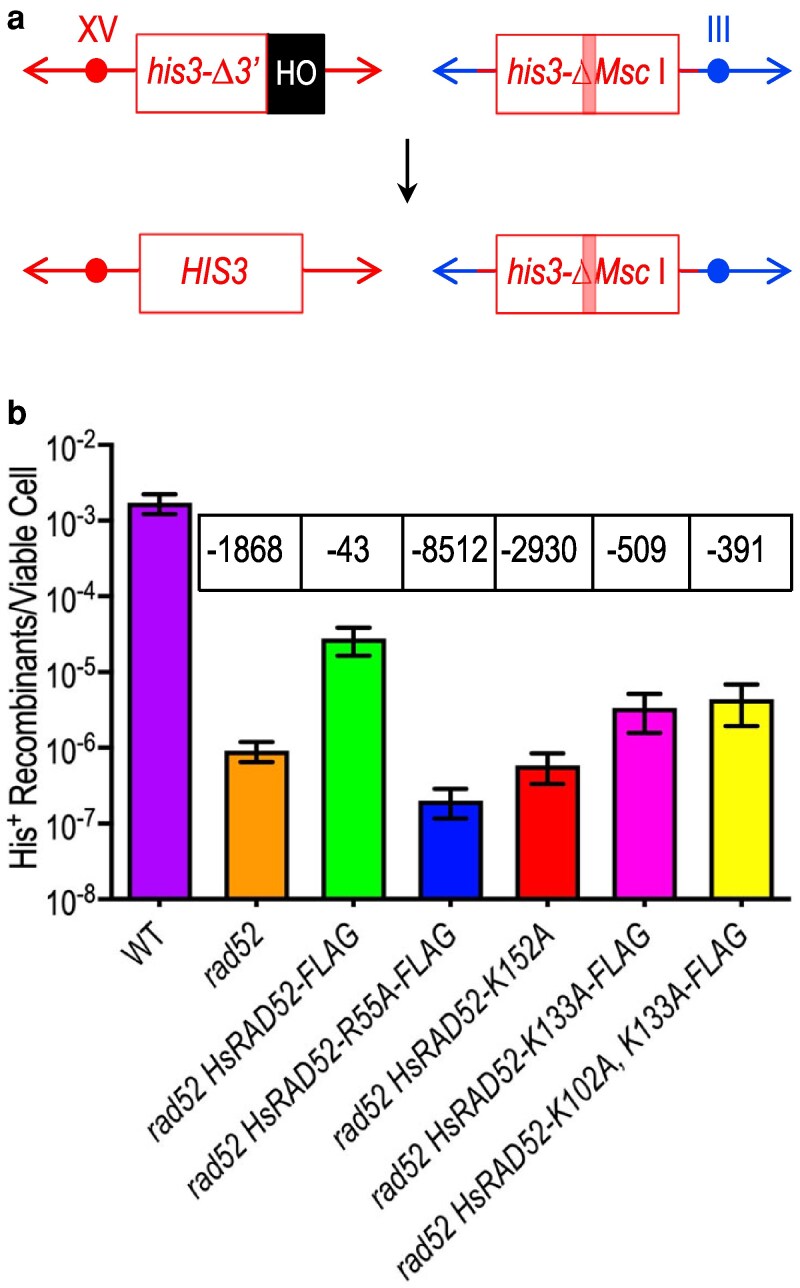
DNA binding mutant alleles of *adh1::HsRAD52-FLAG* are defective in the suppression of the loss of DSB repair by conservative HRR in *rad52*-null mutant budding yeast cells. a) Cartoon depicting DSB-stimulated *his3* EGC assay The *his3-Δ3*′*-HOcs* substrate (“*his3Δ-3*′ box) at the *HIS3* locus on chromosome XV substitutes an HO cut site (“HO” box) for the 3′ end of the *HIS3* coding sequence and flanking DNA. The *his3-ΔMsc* I substrate (“*his3-ΔMsc* I” box) proximal to the *LEU2* locus on chromosome III is comprised of the *HIS3* gene disrupted by the insertion of a *Not* I linker into the *Msc* I site (pink bar). Repair of a HO-catalyzed DSB at the *his3-Δ3*′*-HOcs* substrate by unidirectional transfer of information from the *his3-ΔMsc* I substrate (downward arrow) creates an intact *HIS3* gene. b) The DNA binding mutant alleles of *HsRAD52* confer defects in EGC Expression of HO-endonuclease was induced in cultures of wild-type, *rad52*Δ, *rad52Δ HsRAD52-FLAG*, *rad52Δ HsRAD52-R55A-FLAG*, *rad52Δ HsRAD52-K152A*, *rad52Δ HsRAD52-K133A*, and *rad52Δ HsRAD52-K103A, K133A* strains carrying the EGC substrates before plating onto YPD medium to determine viability or medium lacking histidine to select for recombinants. Colonies were counted and frequencies of EGC determined by dividing the number of His^+^ recombinants by the number of viable cells plated. Mean frequencies of EGC and 95% confidence intervals were plotted. Fold differences below (−) wild-type are in boxes above the plot of each mean frequency.

HRR mechanisms that conserve genome structure (Cejka et al. 2010) have been implicated in the repair of both ionizing radiation- and megaendonuclease-induced DSBs by HsRAD52 in mammalian and budding yeast cells (Park 1995; Feng et al. 2011; Manthey et al. 2017; Clear et al. 2020). Accordingly, HsRAD52 forms foci in mammalian cells exposed to ionizing radiation (Feng et al. 2011) and associates with recombination substrates during repair of HO-endonuclease catalyzed DSBs in the genome of budding yeast cells (Manthey et al. 2017; Clear et al. 2020), suggesting that HsRAD52 binds DNA during DSB repair in vivo. We used the *HsRAD52* DNA binding mutant alleles to investigate the relationship between the DNA binding activity of HsRAD52 and its function in the repair of an HO endonuclease-catalyzed genomic DSB by EGC, a conservative mechanism of HRR (Fig. 3a). The *HsRAD52-R55A* and *-K152A* alleles were chosen to examine the effect of mutating conserved amino acids that contribute to a positively charged, internal groove formed upon multimerization of HsRAD52 (Kagawa et al. 2002; Singleton et al. 2002; Liang et al. 2024), and required for a ssDNA binding activity in vitro (Kagawa et al. 2002; Saotome et al. 2018; Liang et al. 2024). The *HsRAD52-K133A* and *-K102A, K133A* alleles were used to investigate the effect of altering conserved amino acids implicated in the structure of a second, discrete, external DNA binding domain (Kagawa et al. 2008; Saotome et al. 2018; Liang et al. 2024) as well as annealing and D-loop formation with complementary DNA molecules in vitro (Kagawa et al. 2008; Saotome et al. 2018).

As observed previously (Clear et al. 2020), expression of a wild-type *HsRAD52* allele substantially suppresses the profound defect in DSB-stimulated EGC in *rad52-*null mutant yeast cells (Fig. 3b, Supplementary Table 2), consistent with HsRAD52 supporting conservative HRR in budding yeast (Manthey et al. 2017; Clear et al. 2020). Substituting the wild-type allele with the internal DNA binding domain mutant alleles *HsRAD52-R55A* or *-K152A* led to a complete loss of this suppression, with frequencies of EGC that were 1.6- to 4.6-fold below the levels observed in *rad52-*null mutant cells (*P* ≤ 0.0373). In contrast, expression of the external DNA binding domain mutant alleles *HsRAD52-K133A* and *-K102A, K133A* led to frequencies of EGC that were 3.7- to 4.8-fold above the levels observed in *rad52-*null mutant cells (*P* < 0.0001), although approximately 10-fold below the frequency supported by the wild-type allele. These results indicate that while altering either DNA binding domain disrupts the function of HsRAD52 in conservative HRR, altering the internal DNA binding domain disrupts its function to a significantly greater extent than altering the external DNA binding domain.

### Mutations altering the internal DNA binding domain but not the external DNA binding domain of HsRAD52 disrupt its function in the repair of a genomic DSB by non-conservative HRR in budding yeast cells

HsRAD52 can facilitate the repair of a DSB between non-tandem, directly repeated genomic sequences by DRR, producing chromosomal deletions in mammalian and budding yeast cells (Honma et al. 1997; Clear et al. 2020). In contrast to EGC, DRR cannot preserve genome structure and displays distinct genetic control (Ivanov et al. 1996; Tsukamoto et al. 2003; Stark et al. 2004; Clear et al. 2020). Models for DRR propose that exonucleolytic processing of a DSB between repeated genomic sequences reveals complementary single-stranded sequences whose annealing into a double-stranded intermediate is an obligatory step in repair (Cejka et al. 2010). The ability of HsRAD52 to bind and anneal complementary ssDNAs *in vitro* (Kagawa et al. 2008), and disruption of these activities by mutations altering the internal and external DNA binding domains (Kagawa et al. 2002, 2008; Saotome et al. 2018; Liang et al. 2024) suggests these domains may play a role in the repair of a DSB by DRR in budding yeast.

**Fig. 4.**
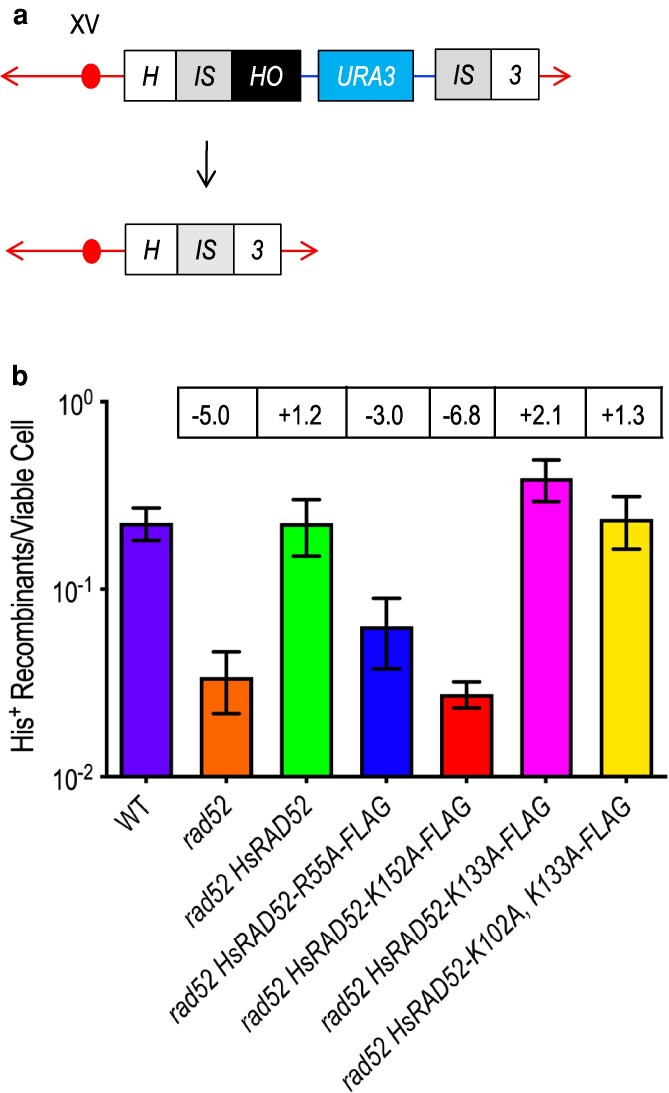
The internal and external DNA binding domain mutant alleles of *HsRAD52-FLAG* have distinct effects on DSB repair by non-conservative HRR in *rad52-*null mutant budding yeast cells. a) Cartoon depicting DSB repair by recombination between non-tandem direct repeats At the *HIS3* locus on chromosome XV, DSB formation by HO endonuclease at a HO cut site inserted at the centromere distal edge of the centromere proximal duplication of a segment of the *HIS3* coding sequence (left *IS* box) initiates recombination between the centromere proximal and distal repeats (left and right *IS* boxes) while deleting intervening plasmid sequences (blue line and *URA3* marker box) and creating an intact *HIS3* gene. b) The internal and external DNA binding domain mutant alleles of *adh1::HsRAD52-FLAG* have distinct effects on DSB repair by DRR Expression of HO-endonuclease was induced in cultures of wild-type, *rad52Δ*, *rad52Δ HsRAD52-FLAG*, *rad52Δ HsRAD52-R55A-FLAG*, *rad52Δ HsRAD52-K152A*, *rad52Δ HsRAD52-K133A*, and *rad52Δ HsRAD52-K103A, K133A* strains containing the DRR substrate before plating onto YPD medium to determine viability, and onto medium lacking histidine to select for recombinants. Colonies were counted and frequencies of DRR determined by dividing the number of His^+^ recombinants by the number of viable cells plated. Mean frequencies of DRR and 95% confidence intervals were plotted against genotype. Fold differences above (+) and below (−) wild-type are in boxes above the plot of each mean frequency.

The *HsRAD52* DNA binding mutant alleles were used to study the potential role of the internal and external DNA binding domains of HsRAD52 in the repair of an HO endonuclease-catalyzed genomic DSB by DRR (Fig. 4a). As observed previously (Clear et al. 2020), the expression of a wild-type *HsRAD52* allele in *rad52-*null mutant budding yeast cells supported a wild-type level of DRR (*P* = 0.9825, Fig. 4b, Supplementary Table 2), indicating that HsRAD52 suppresses the loss of DRR conferred by the *rad52-*null mutation. In keeping with the deleterious effects of the mutations on DNA binding by HsRAD52 *in vitro* (Kagawa et al. 2002, 2008; Saotome et al. 2018) we observed that expressing the internal DNA binding domain mutant alleles *HsRAD52-R55A* and *-K152A* in *rad52-*null mutant budding yeast cells supported frequencies of DRR that were 3.0- and 6.8-fold below, and significantly different from those observed in wild-type cells (*P* < 0.0001). Interestingly, while the frequency of DRR supported by the *-K152A* allele was not significantly different from the frequency observed in *rad52-*null mutant cells (*P* = 0.5264), the frequency supported by *-R55A* was significantly higher than the frequencies observed in *rad52-*null mutant cells, either with or without the *-K152A* allele (*P* ≤ 0.0068). These results indicate that these mutations significantly but differentially reduce DRR in budding yeast.

In striking contrast to the internal DNA binding domain mutant alleles, expression of the external DNA binding domain mutant allele *HsRAD52-K133A* in *rad52-*null mutant cells supported a frequency of DRR 2.1-fold greater and significantly different from the frequency observed in wild-type budding yeast cells (*P* = 0.006, Fig. 4b, Supplementary Table 2). Expression of the -*K102A, K133A* allele supported a frequency of DRR that was 1.3-fold greater than but not significantly different from the wild-type frequency (*P* = 0.6135). These results indicate that these mutations do not reduce DRR in budding yeast.

### Modeling the impact of DNA binding mutants on the HsRAD52 protein-DNA interfaces

The impact of the *HsRAD52-R55A, -K152A, -K133A,* and *-K102A, K133A* mutations on DSB repair by HsRAD52 in budding yeast, prompted a further investigation of the effects of these mutations on the atomic-level interactions with ssDNA. We used the previously published X-ray structures of ssDNA bound to the internal and external DNA binding domains of HsRAD52 (PDB 5XRZ and PDB 5XS0, respectively) (Saotome et al. 2018) to model how the DNA binding mutations affect the protein-DNA interfaces of the mutant proteins. Amino acids R55 and K152 form hydrogen bonds that stabilize the binding of ssDNA to the internal DNA binding domain (Supplementary Fig. 1a and b). Amino acids K102 and K133 occupy the external DNA binding domain with K102 forming a hydrogen bond with ssDNA (Supplementary Fig. 1c and d). Visualization of the calculated electrostatic surface potentials of the wild-type and mutant HsRAD52 structures revealed effects of the mutations on the shapes of the DNA binding domains (Supplementary Fig. 1e and f). The R55A and K152A amino acid changes reduced the positive charge of the surface of the internal DNA binding domain with K152A having the most pronounced effect on the charge and shape of the binding pocket. The K133A and K102A, K133A amino acid changes reduced the positive charge of the outer DNA binding site and altered the surface shape. These observations are consistent with the changes in the capacity of HsRAD52 to support conservative DSB repair exerted by these mutations in budding yeast (Fig. 3, Supplementary Table 2).

### Occurrence of mutations in analyzed cancer genomes

Previous studies using budding yeast and mammalian cell models have associated loss of HsRAD52 DSB repair function with changes in cancer susceptibility in humans (Adamson et al. 2020; Clear et al. 2020). This motivated our investigation of a potential linkage of the *HsRAD52-R55A, -K152A, -K102A* and *-K133A* DNA binding mutations with cancer susceptibility. We queried the COSMIC and cBioPortal databases (Cerami et al. 2012; Gao et al. 2013; Sondka et al. 2024) to investigate whether the *HsRAD52* DNA binding mutations appear in cancer genomes but found that they did not appear among the reported *HsRAD52* mutations. The reported frequency of samples with any *HsRAD52* mutations was 0.78% in COSMIC (511 unique samples out of 65,519 total queries) and 0.28% in cBioPortal (271 unique samples out of 94,378 total queries). Significantly, our previous study identified an -*R55H* substitution mutation at this locus (Hamid et al. 2022). When we extracted information for all *-R55H* samples, we found that it occurs in three of the COSMIC samples (0.59%) and six of the cBioPortal samples (2.2%) that carry *HsRAD52* mutations (Supplementary Table 3). The cBioPortal *-R55H* mutation frequency is consistent with a mutation hotspot (Gao et al. 2017; Ghandi et al. 2019; Juul et al. 2021). The difference between the COSMIC and cBioPortal frequencies is due to different patient sources, with only one of the samples (73y female) shared between the two repositories. Analyses using two algorithms predictive of functional pathogenicity (CHASM and VEST) (Carter et al. 2013; Douville et al. 2016; Tokheim and Karchin 2019) showed that *-R55H* is likely to be pathogenic (*P* = 0.0028) but not to be a core driver mutation (*P* = 0.0989) (Supplementary Table 3). The study also revealed that *-R55H* can co-occur with *BRCA1* and *BRCA2* mutations. One COSMIC-reported sample (Tumor metastasized to the liver) had two *BRCA1* driver mutations (P871L, *P* = 0.0002; and K1183R, *P* = 0.0053) and one *BRCA2* driver mutation (N372H, *P* = 0.0472). A cBioPortal-reported sample (74y female) had two *BRCA1* driver mutations (L1306F, *P* = 0.0069; K748T, *P* = 0.0055) and one *BRCA2* driver mutation (N1713H, *P* = 0.0414). These calculations did not include the S2372* truncation mutation that deletes the part of the C-terminus encoding the DNA binding domain and the nuclear localization sequence, rendering the protein non-functional. While the zygosity of -*R55H* in the tumors is unknown, these results indicate that the presence of *-R55H* does not elicit the synthetic lethality in *BRCA*-defective human tumors that has been observed when RAD52 is depleted in *BRCA-*defective cells in vitro (Feng et al. 2011; Lok and Powell 2012; Lok et al. 2013). Accordingly, an analysis using the cBioPortal mutual exclusivity calculator shows that there is a statistically significant tendency for *HsRAD52* missense mutations appearing in the database to co-occur with missense mutations in either *BRCA1* or *BRCA2* in human cancers (*P* < 0.001).

## Discussion

HsRAD52 possesses discrete DNA binding activities that are associated with internal and external domains of the HsRAD52 multimer that facilitate the formation of structurally and biochemically distinct complexes with DNA (Supplementary Fig. 1) (Kagawa et al. 2002, 2008; Saotome et al. 2018; Liang et al. 2024). Mutations altering conserved amino acids that change the charge and shape of these domains (Supplementary Fig. 1e and f) diminish several biochemical activities *in vitro* (Kagawa et al. 2008; Saotome et al. 2018). We previously demonstrated that HsRAD52 can associate with DSBs in *rad52-*null mutant yeast cells while facilitating their repair by HRR (Manthey et al. 2017; Clear et al. 2020), suggesting the suitability of this system for studying the effects of these mutations on HRR *in vivo*.

Expression of a wild-type *HsRAD52* allele significantly suppresses the severe defect in the repair of a HO-endonuclease catalyzed DSB by conservative HRR in *rad52-*null mutant yeast cells (Fig. 3, Supplementary Table 3) (Manthey et al. 2017; Clear et al. 2020), confirming that HsRAD52 supports conservative DSB repair in this *in vivo* model system. In contrast, expression of the internal DNA binding domain mutant alleles did not suppress this DSB repair defect (Fig. 3, Supplementary Table 2). This loss of function *in vivo* correlates with the defects displayed by HsRAD52-R55A and -K152A in ssDNA binding *in vitro* (Kagawa et al. 2002; Saotome et al. 2018), an activity hypothesized to be fundamental to the function of HsRAD52 in the repair of genomic DSBs by conservative HRR in human and budding yeast cells (Manthey et al. 2017; Clear et al. 2020; Muhammad et al. 2024). These results suggest that anchoring HsRAD52 multimers to the phosphate backbone of the DNA by arginine 55 and the stabilization of a B-form conformation of the DNA by lysine 152 (Supplementary Fig. 1) (Saotome et al. 2018; Liang et al. 2024) provide a key intermediate in DSB repair by conservative HRR in this system.

**Fig. 5.**
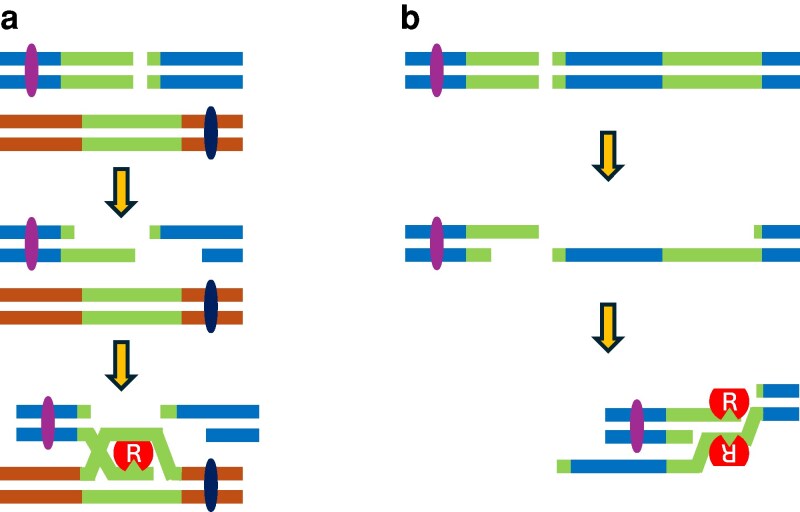
Speculative model for the action of HsRAD52 in repair of a chromosomal DSB by conservative and non-conservative HRR in budding yeast. a) HsRAD52 action in conservative HRR A DSB in one copy of a duplicate genomic sequence initiates exonucleolytic processing that generates ssDNA at the broken ends. HsRAD52 (red ovoid) binds to the ssDNA via the internal DNA binding site (triangular cutout) and facilitates D-loop formation with the unlinked, intact duplicate sequence. HsRAD52 binds to the displaced strand of the intact repeat via the external DNA binding site (flat surface), stabilizing the D-loop intermediate. b) HsRAD52 action in non-conservative HRR A DSB between non-tandem duplicate genomic sequences initiates exonucleolytic processing that generates complementary ssDNA sequences at both broken ends. HsRAD52 bound to the complementary ssDNA sequences via the internal DNA binding site mediates annealing into a dsDNA intermediate.

In addition to disrupting ssDNA binding the external DNA binding domain mutations also impart defects in dsDNA binding, supercoiling and D-loop formation *in vitro* (Supplementary Fig. 1f) (Kagawa et al. 2002, 2008; Saotome et al. 2018), which could impinge upon the ability of HsRAD52 to support heteroduplex formation during conservative HRR in humans and budding yeast (Manthey et al. 2017; Saotome et al. 2018; Clear et al. 2020). These mutations are also hypothesized to disrupt the networks formed by DNA binding to the external DNA binding domains of multiple HsRAD52 ring structures that may be important for the annealing of complementary DNA sequences (Saotome et al. 2018). Consistent with the importance of these biochemical activities, expression of the external DNA binding domain mutant alleles in *rad52-*null mutant yeast cells led to significantly reduced levels of DSB repair by conservative HRR (Fig. 3, Supplementary Table 2). Interestingly, while indicative of substantial dysfunction, the frequencies of conservative HRR supported by the external DNA binding domain mutant alleles were significantly higher than those supported by expression of the internal DNA binding domain mutant alleles. These observations suggest that the biochemical and structural defects conferred by the internal and external DNA binding domain mutations exert distinct effects on conservative DSB repair by HsRAD52 in budding yeast (Fig. 5). Further, these results suggest that, in this model system, the anchoring and stabilization of the association of DNA with HsRAD52 mediated by the internal binding domain may be of greater importance than the chambering and simultaneous trapping of multiple DNA molecules supported by the external binding domain (Saotome et al. 2018).

As reported previously (Clear et al. 2020), expression of the wild-type *HsRAD52* allele in a *rad52-*null mutant strain completely suppresses its defect in DSB repair by non-conservative HRR (Fig. 4, Supplementary Table 3), indicating that HsRAD52 can functionally replace the budding yeast RAD52 protein in non-conservative HRR in this model system. Like their effect on conservative HRR, expression of the *HsRAD52* internal DNA binding domain mutant alleles did not suppress the loss of non-conservative HRR in *rad52-*null mutant yeast strains. These *in vivo* observations are mirrored by the reduced levels of ssDNA annealing that the internal DNA binding mutant proteins support *in vitro* (Saotome et al. 2018), suggesting that these activities are analogous, and dependent on the formation and stabilization of the interaction between ssDNA and the internal HsRAD52 binding domain. Interestingly, the significantly lower level of non-conservative HRR observed when expressing *HsRAD52-K152A* than *-R55A* is reflected by a similar difference in the levels of ssDNA annealing produced by the mutant proteins *in vitro* (Fig. 4, Supplementary Table 2) (Saotome et al. 2018). This suggests that the anchoring of DNA facilitated by lysine 152 may be more critical for ssDNA annealing *in vitro* and non-conservative HRR *in vivo* than the stretching of the phosphate backbone of DNA facilitated by arginine 55 providing further support for HsRAD52 having overlapping functionality in ssDNA annealing *in vitro* and non-conservative HRR in the budding yeast model system.

Crystallographic and cryo-electron microscopy data have documented stable interactions between ssDNA and lysines 103 and 133 of the external DNA binding domain of HsRAD52 (Saotome et al. 2018; Liang et al. 2024) that support the annealing of complementary ssDNA strands *in vitro* (Kagawa et al. 2008; Saotome et al. 2018). Paradoxically, expression of the external DNA binding domain mutant alleles of *HsRAD52* fully suppressed the non-conservative HRR defect in *rad52-*null mutant budding yeast cells (Fig. 4, Supplementary Table 2), indicating no loss of the ability to support DSB repair by non-conservative HRR in this model system. This reveals that the external DNA binding domain of HsRAD52 plays distinct roles in ssDNA annealing *in vitro* and non-conservative HRR in this *in vivo* model system. Further, the ability of the external DNA binding domain mutant alleles to fully suppress the non-conservative but minimally suppress the conservative HRR defect of *rad52-*null mutant budding yeast cells (Fig. 3, Supplementary Table 2) indicates that they are separation of function mutations. This, in turn indicates that the external DNA binding domain of HsRAD52 plays distinct roles in conservative and non-conservative HRR in this model system (Fig. 5).

This study joins a growing body of investigation establishing the utility of budding yeast for studying the function of human DNA repair proteins in a model *in vivo* system (Caligo et al. 2009; Guaragnella et al. 2014; Maresca et al. 2018; Belle et al. 2022; Lodovichi et al. 2022; Law et al. 2023, 2024; Galli et al. 2024). Examining the impact of mutations in the human DNA repair genes that confer defined biochemical defects on protein function on DSB repair in budding yeast, presents an opportunity to identify and explore correlations between protein function *in vitro* and in an intact organism. Such studies acquire added significance when they lead to correlations between loss of DSB repair *in vivo* and human disease (Adamson et al. 2020; Clear et al. 2020). Intriguingly, the cancer-associated *HsRAD52-R55H* mutation that results in a different amino acid substitution at the same position in the internal ssDNA binding site as the *-R55A* mutation is likely to be pathogenic and co-occurs in tumors bearing mutations in *BRCA1* and *BRCA2* (Supplementary Table 3). Additionally, the statistically significant co-occurrence of missense mutations in *HsRAD52* with mutations in *BRCA1* and *BRCA2* in human cancers suggests that investigating the effects of other disease-associated *HsRAD52* mutations on DSB repair *in vivo* might reveal other parallels between cellular function and disease.

This approach is also poised to contribute to the identification of targets on human DNA repair proteins for small molecule inhibitors, which could be used to develop drugs. For instance, the current studies indicate that disruption of the internal and external DNA binding domains of HsRAD52 exert distinct inhibitory effects on DSB repair by HRR in vivo, suggesting that targeting either one or both might lead to more potent inhibition, and ultimately the development of drug cocktails that more effectively kill HR-defective cancer cells (Hengel et al. 2016; Sullivan et al. 2016; Bhat et al. 2022). Further, the availability in budding yeast of tools to study the association of HsRAD52 with DSBs during their repair, which has been used to quantitate decreases in the association of mutant HsRAD52 proteins with HR substrates (Manthey et al. 2017; Clear et al. 2020), could be used to quantitate the effects of candidate inhibitors at the DNA level *in vivo*. Linked with high-throughput screening, these tools could speed the identification and refinement of viable inhibitors.

## Supplementary Material

jkaf282_Supplementary_Data

## Data Availability

All budding yeast strains and plasmids are available upon request. We affirm that all data required to support the conclusions presented in the article can be viewed within the text, figures, and tables of the article. Supplemental material available at GENETICS online.
