# Supplementary material for: Mutations altering the DNA binding domains of the human RAD52 protein exert distinct effects on homologous recombination repair in Saccharomyces cerevisiae
Source: G3 (Bethesda). 2025 Nov 23;16(2):jkaf282. doi: 10.1093/g3journal/jkaf282 (PMC12869081; doi:10.1093/g3journal/jkaf282)
Supplement: jkaf282_Supplementary_Data [file jkaf282_supplementary_data.docx]

**
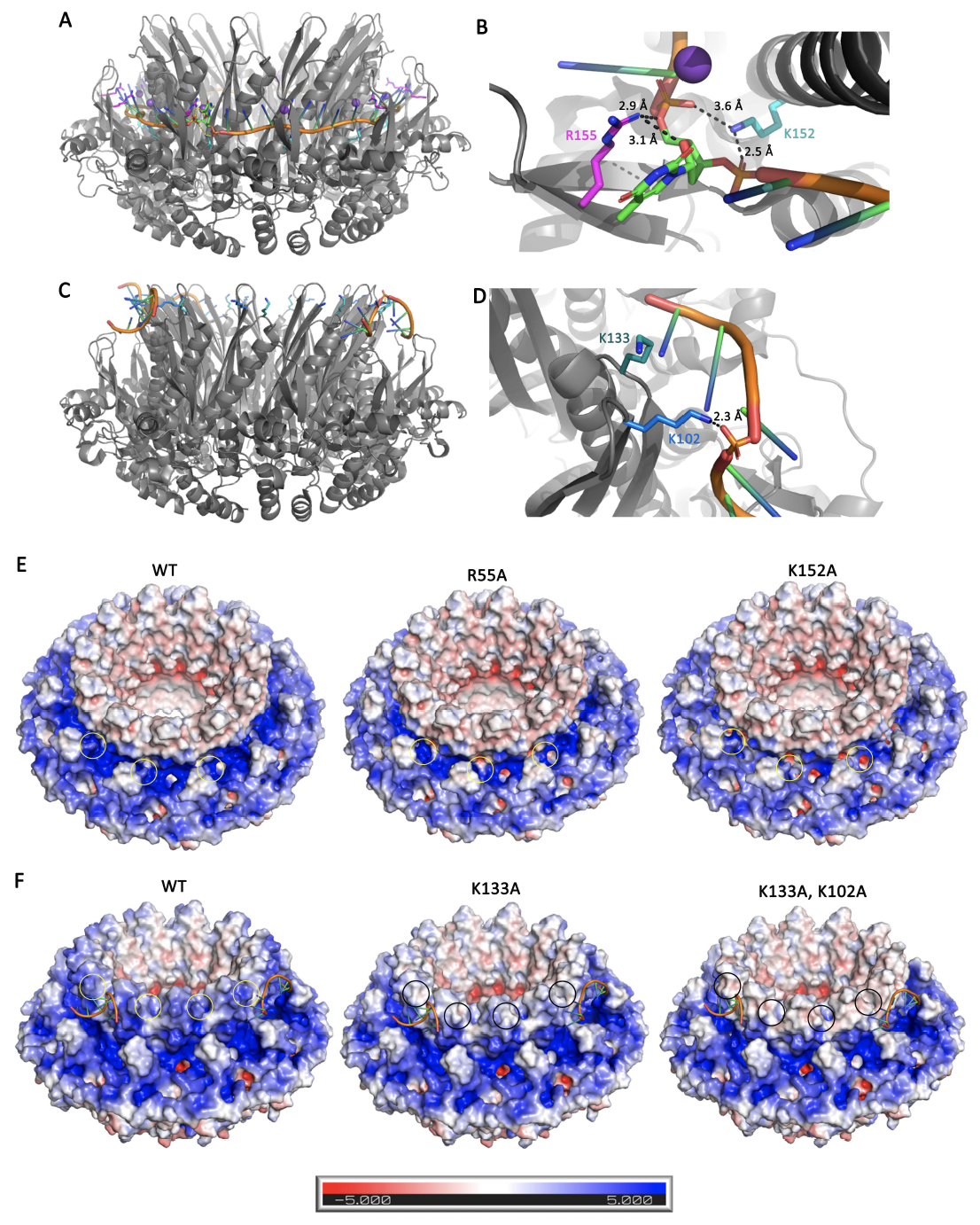
**

**Supplementary Figure** 1 **– Structural analysis of HsRAD52 DNA binding mutants**

1. The structure of HsRAD52 (PDB 5xRZ) is shown in gray with ssDNA (orange backbone) and K^+^ ions (purple spheres) bound to the inner binding site. The side chains of R55 and K152 are shown as magenta and cyan sticks, respectively.
2. The side chain of R55 (magenta sticks) forms two hydrogen bonds (black dashed lines) to the ssDNA molecule, one to an oxygen in the phosphodiester bond and one to a carbonyl oxygen of the thymidine base (green sticks). The side chain of K152 (cyan sticks) forms two hydrogen bonds (black dashed lines) with the phosphodiesters of the ssDNA backbone.
3. The structure of HsRAD52 (PDB 5XS0) is shown in gray with ssDNA (orange backbone). The side chains of K102 and K133 are shown and blue and teal sticks, respectively.
4. The side chain of K102 (blue sticks) forms one hydrogen bond (black dashed line) with the phosphodiester of the ssDNA molecule. The side chain of K133 (teal sticks) is located nearby but does not hydrogen bond with the ssDNA molecule.
5. The electrostatic surface potential of the WT, R55A, and K152A structures are shown. The locations of the R55 and K152 residues are shown with yellow circles.
6. The electrostatic surface potential of the WT, K133A, and K133A/K102A structures are shown. The locations of the K133A and K102 residues are shown with yellow or black circles. The electrostatic potential coloring legend is shown below with red as -5.0 kT/e and blue as +5.0 kT/e.

**Supplementary Table 1 – Y2H Data**

| **Plasmids** | **ß-Galactosidase Activity (Miller Units)** |
| --- | --- |
| pGAD-424/pGBT9 | 0.010  (0.008, 0.012) ^(a)^  [1] |
| pGAD-424/pGBT9-HsRAD52 | 0.008  (0.005, 0.011)  [-1.3] ^(b)^ |
| pGAD-424-HsRAD52/pGBT9 | 0.012  (0.006, 0.017)  [+1.2] |
| pGAD-424-HsRAD52/  pGBT9-HsRAD52 | 0.795  (0.642, 0.948)  [+80] |
| pGAD-424-HsRAD52-R55A/  pGBT9-HsRAD52-R55A | 0.330  (0.227,0.433)  [+33] |
| pGAD-424-HsRAD52-K152A/  pGBT9-HsRAD52-K152A | 0.314  (0.179, 0.450)  [+31] |
| pGAD-424-HsRAD52-K133A/  pGBT9-HsRAD52-K133A | 0.828  (0.432, 1.22)  [+83] |
| pGAD-424-HsRAD52-K102A, K133A/  pGBT9-HsRAD52-K102A, K133A | 0.498  (0.392, 0.605)  [+50] |

^(a)^ 95% confidence interval

^(b)^ Fold difference from negative control

**Supplementary Table 2 - HRR Data**

| **Genotype** | **DSB-EGC ^(a)^** | **DSB-DRR ^(b)^** |
| --- | --- | --- |
| WT | 1.72x10^-3 (c)^  (1.37, 2.07) ^(d)^  [1] | 1.88x10^-1 (e)^  (1.25, 2.51)  [1] |
| *rad52∆* | 9.21x10^-7 (c)^  (6.52, 11.90)  [-1868] ^(f)^ | 3.80x10^-2 (e)^  (2.35, 5.26)  [-5.0] |
| *rad52∆ HsRAD52* | 7.20x10^-5 (c)^  (5.73, 8.68)  [-24] | 2.28x10^-1 (e)^  (1.50. 3.01)  [+1.2] |
| *rad52∆ HsRAD52-FLAG* | 3.96x10^-5 (c)^  (1.13, 6.79)  [-43] | n.d.^(g)^ |
| *rad52∆ HsRAD52-R55A-FLAG* | 2.02x10^-7^  (1.17, 2.86)  [-8512] | 6.37x10^-2^  (3.77, 8.97)  [-3.0] |
| *rad52∆ HsRAD52-K152A-FLAG* | 5.87x10^-7^  (3.21, 8.43)  [-2930] | 2.77x10^-2^  (2.33, 3.22)  [-6.8] |
| *rad52∆ HsRAD52-K133A-FLAG* | 3.38x10^-6^  (1.57, 5.18)  [-509] | 3.93x10^-1^  (2.94, 4.91)  [+2.1] |
| *rad52∆ HsRAD52-K102A, K133A-FLAG* | 4.40x10^-6^  (2.47, 9.65)  [-391] | 2.39x10^-1^  (1.64, 3.13)  [+1.3] |

^(a)^ Double-strand Break Stimulated Ectopic Gene Conversion Frequency (recombinants/viable cell)

^(b)^ Double-strand Break Stimulated Direct Repeat Recombination Frequency (recombinants/viable cell)

^(c)^ Manthey et al, 2017

^(d)^ 95% confidence interval

^(e)^ Clear et al., 2020

^(f)^ Fold difference from wild-type

^(g)^ Not Determined

**Supplementary Table 3 - BRCA1 and BRCA2 mutations co-occurring with RAD52 R55H**

**
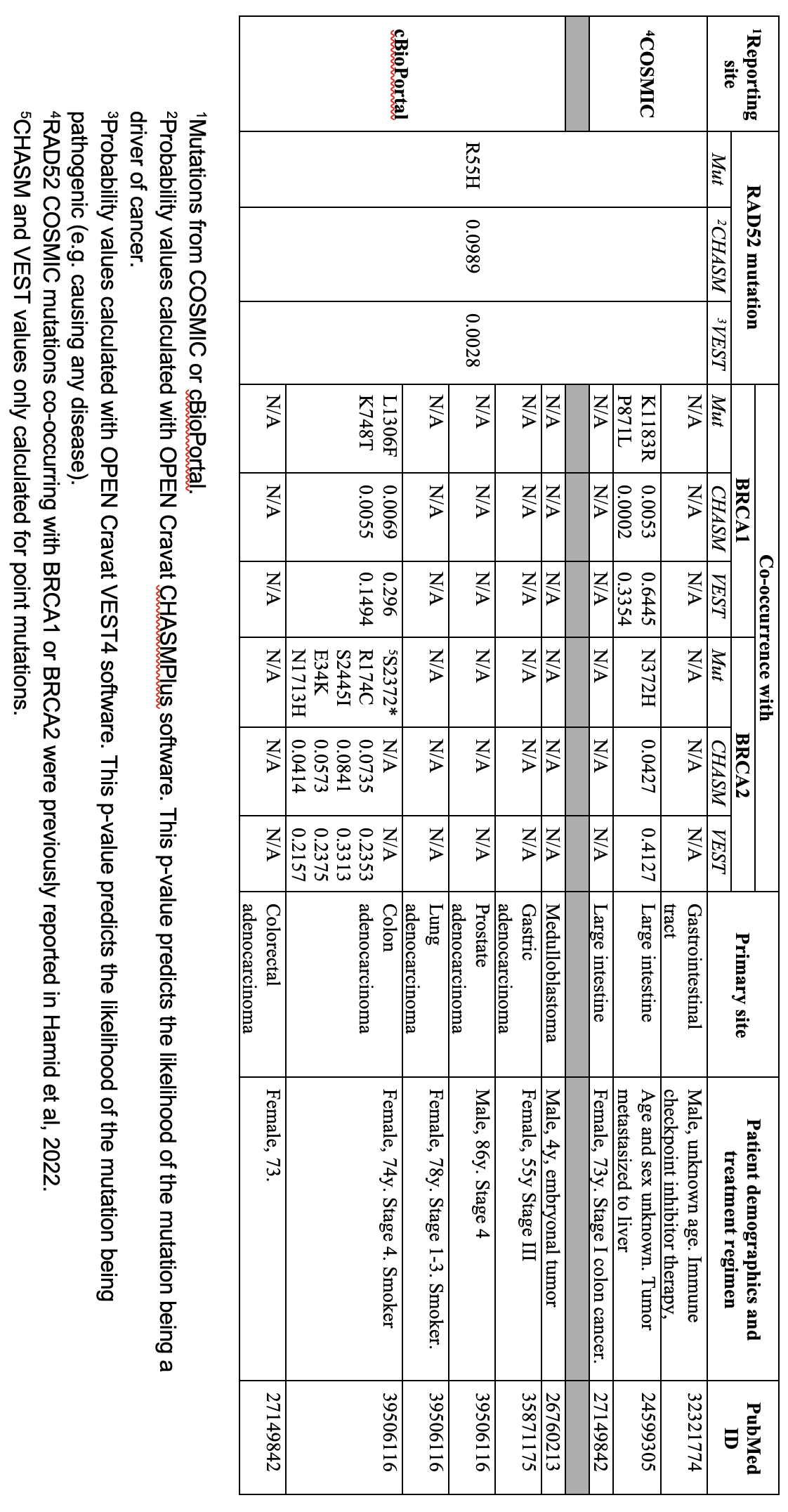
**

**Supplementary Table 4 –** *Saccharomyces cerevisiae* strains used in this study

| STRAIN | GENOTYPE ^(^**^a^**^)^ | ASSAY | ORIGIN |
| --- | --- | --- | --- |
| ABX3599 | *MAT****a****/MATα, ADE2/ade2-1, HIS3/his3-11, -15, LEU2/leu2-3, -112, URA3/ura3-1, ADH1/adh1::HsRAD52-FLAG::KANMX::HYGMX::ADH1* | Western | This study |
| ABX3980 | *MAT****a****/MATα, ADE2/ade2-1, HIS3/his3-11, -15, LEU2/leu2-3, -112, URA3/ura3-1, ADH1/adh1::HsRAD52-R55A-FLAG::KANMX::HYGMX::ADH1* | Western | This study |
| ABX3986 | *MAT****a****/MATα, ADE2/ade2-1, HIS3/his3-11, -15, URA3/ura3-1, ADH1/adh1::HsRAD52-K152A-FLAG::KANMX::HYGMX::ADH1* | Western | This study |
| ABX4027 | *MAT****a****/MATα, ADE2/ade2-1, HIS3/his3-11, -15, URA3/ura3-1, ADH1/adh1::HsRAD52-K133A-FLAG::KANMX::HYGMX::ADH1* | Western | This study |
| ABX4024 | *MAT****a****/MATα, ADE2/ade2-1, HIS3/his3-11, 15, URA3/ura3-1, ADH1/adh1::HsRAD52-K103A, K133A-FLAG::KANMX::HYGMX::ADH1* | Western | This study |
| ABX3761 | *MATa::LEU2/MATa::LEU2, his3-∆3'-HOcs/his3-11, -15, LEU2::his3-∆MscI/leu2-3, -112, trp1::GAL-HO-KANMX/trp1-1, RAD52/rad52::TRP1, ADH1/adh1::HsRAD52-FLAG-KAN-MX::HYGMX::ADH1,* pJM3 (*URA3, MAT****a***)*,* pLAY263 *(HIS3, MATα*) | DSB-EGC ^(^**^b^**^)^ | This study |
| ABX3982 | *MATa::LEU2/ MATa::LEU2, his3-∆3'-HOcs/his3-11, -15, LEU2::his3-∆MscI/leu2-3, -112, trp1::GAL-HO-KANMX/trp1-1, RAD52/rad52::TRP1, ADH1/adh1::HsRAD52-R55A-FLAG::KANMX::HYGMX::ADH1,* pJM3 (*URA3, MAT****a***)*,* pLAY263 *(HIS3, MATα*) | DSB-EGC | This study |
| ABX4032 | *MATa::LEU2/MATa::LEU2, his3-∆3'-HOcs/his3-11, -15, LEU2::his3-∆MscI/leu2-3, -112, trp1::GAL-HO-KANMX/trp1-1, RAD52/rad52::TRP1, ADH1/adh1::HsRAD52-K152A::KANMX::HYGMX::ADH1,* pJM3 (*URA3, MAT****a***)*,* pLAY263 *(HIS3, MATα*) | DSB-EGC | This study |
| ABX4045 | *MATa::LEU2/MATa::LEU2, his3-∆3'-HOcs/his3-11, -15, LEU2::his3-∆MscI/leu2-3, -112, trp1::GAL-HO-KANMX/trp1-1, RAD52/rad52::TRP1, ADH1/adh1::HsRAD52-K133A-FLAG::KANMX::HYGMX::ADH1,* pJM3 (*URA3, MAT****a***)*,* pLAY263 *(HIS3, MATα*) | DSB-EGC | This study |
| ABX4036 | *MATa::LEU2/MATa::LEU2, his3-∆3'-HOcs/his3-11, -15, LEU2::his3-∆MscI/leu2-3, -112, trp1::GAL-HO-KANMX/trp1-1, RAD52/rad52::TRP1, ADH1/adh1::HsRAD52-K102A, K133A-FLAG::KANMX::HYGMX::ADH1,* pJM3 (*URA3, MAT****a***)*,* pLAY263 *(HIS3, MATα*) | DSB-EGC | This study |
| ABX3632 | *MATa::LEU2/MAT****a****, ADE2/ade2-1, his3::HOcs::URA3::his3/his3-11, -15, trp1::GAL-HO-KANMX /trp1-1, RAD52/rad52::TRP1, ADH1/adh1::HsRAD52-FLAG::KANMX::HYGMX::ADH1,* pLAY263 *(HIS3, MATα)* | DSB-DRR **^(c)^** | This study |
| ABX4031 | *MATa::LEU2/MAT****a****, ADE2/ade2-1, his3::HOcs::URA3::his3/his3-11, -15, trp1::GAL-HO::KANMX/trp1-1, RAD52/rad52::TRP1, ADH1/adh1::HsRAD52-R55A-FLAG::KANMX::HYGMX::ADH1,* pLAY263 *(HIS3, MATα)* | DSB-DRR | This study |
| ABX3988 | *MATa::LEU2/MAT****a****, ADE2/ade2-1, his3::HOcs::URA3::his3/his3-11, -15, trp1::GAL-HO::KANMX/trp1-1, RAD52/rad52::TRP1, ADH1/adh1::HsRAD52-K152A-FLAG::KANMX::HYGMX::ADH1,* pLAY263 *(HIS3, MATα)* | DSB-DRR | This study |
| ABX4043 | *MATa::LEU2/MAT****a****, ADE2/ade2-1, his3::HOcs::URA3::his3/his3-11, -15, leu2-3, 112/leu2-3, -112, trp1::GAL-HO::KANMX/trp1-1, RAD52/rad52::TRP1, ADH1/adh1:HsRAD52-K133A-FLAG:KANMX:HYGMX::ADH1,* pLAY263 *(HIS3, MATα),* | DSB-DRR | This study |
| ABX4035 | *MATa::LEU2/MAT****a****, ADE2/ade2-1, his3::HOcs::URA3::his3/his3-11, 15, trp1::GAL-HO::KANMX/trp1-1, RAD52/rad52::TRP1, ADH1/adh1::HsRAD52-K102A, K133A::KANMX::HYGMX::ADH1,* pLAY263 *(MATα , HIS3)* | DSB-DRR | This study |
| Y187 | *MATα, ade2-101, his3-∆200, leu2-3, 112, trp1-901, URA3::GAL1_UAS_-GAL1_TATA_-lacZ, gal4Δ, gal80Δ, met^-^* | Y2H **^(d)^** | Clontech |

^(^**^a^**^)^ All strains except Y187 are isogenic and possess the following genotype unless otherwise

noted: *ade2-1/ade2-1, can1-100/can1-100, his3-11, -15/his3-11, -15, leu2-3, -112/leu2-3, -112,*

*trp1-1/trp1-1, ura3-1/ura3-1*

^(^**^b^**^)^ Double-strand break stimulated ectopic gene conversion

**^(c)^** Double-strand break stimulated direct repeat recombination

**^(d)^** Yeast two-hybrid

**Supplementary Table 5 –** Plasmids used in this study

| NAME | DESCRIPTION | SOURCE |
| --- | --- | --- |
| pGBT9 | Multi-copy bacterial-yeast shuttle vector containing a *TRP1* selectable marker and the coding sequence of Gal4 DNA binding domain | Clontech |
| pGBT9-HsRAD52 | Derivative of pGBT9 for expression of wild-type HsRAD52-Gal4 DNA binding domain fusion | Manthey et al., 2017 |
| pGBT9-HsRAD52-R55A | Derivative of pGBT9-HsRAD52 for expression of mutant HsRAD52-R55A-Gal4 DNA binding domain fusion | This study |
| pGBT9-HsRAD52-K152A | Derivative of pGBT9-HsRAD52 for expression of mutant HsRAD52-K152A-Gal4 DNA binding domain fusion | This study |
| pGBT9-HsRAD52-K133A | Derivative of pGBT9-HsRAD52 for expression of mutant HsRAD52-K152A-Gal4 DNA binding domain fusion | This study |
| pGBT9-HsRAD52-K103A, K133A | Derivative of pGBT9-HsRAD52 for expression of mutant HsRAD52-K103A, K133A-Gal4 DNA binding domain fusion | This study |
| pGAD424 | Multi-copy bacterial-yeast shuttle vector containing a *LEU2* selectable marker and the coding sequence of Gal4 transcription activation domain | Clontech |
| pGAD424-HsRAD52 | Derivative of pGAD424 for expression of wild-type HsRAD52-Gal4 transcription activation domain fusion | Manthey et al., 2017 |
| pGAD424-HsRAD52-R55A | Derivative of pGAD424-HsRAD52 for expression of mutant HsRAD52-R55A-Gal4 transcription activation domain fusion | This study |
| pGAD424-HsRAD52-K152A | Derivative of pGAD424-HsRAD52 for expression of mutant HsRAD52-K152A-Gal4 transcription activation domain fusion | This study |
| pGAD424-HsRAD52-K133A | Derivative of pGAD424-HsRAD52 for expression of mutant HsRAD52-K133A-Gal4 transcription activation domain fusion | This study |
| pGAD424-HsRAD52-K103A, K133A | Derivative of pGAD424-HsRAD52 for expression of mutant HsRAD52-K103A, K133A-Gal4 transcription activation domain fusion | This study |
